# Inhibition of Ultraviolet B-Induced Expression of the Proinflammatory Cytokines TNF-α and VEGF in the Cornea by Fucoxanthin Treatment in a Rat Model

**DOI:** 10.3390/md14010013

**Published:** 2016-01-07

**Authors:** Shiu-Jau Chen, Ching-Ju Lee, Tzer-Bin Lin, Hsiang-Jui Liu, Shuan-Yu Huang, Jia-Zeng Chen, Kuang-Wen Tseng

**Affiliations:** 1 Department of Neurosurgery, Mackay Memorial Hospital, Taipei 10449, Taiwan; chenshiujau@gmail.com; 2 Internal Medicine, Taipei Hospital, Ministry of Health and Welfare, New Taipei 24213, Taiwan; lululee66@yahoo.com.tw; 3 Department of Physiology, School of Medicine, College of Medicine, Taipei Medical University, Taipei 11049, Taiwan; tblin2@tmu.edu.tw; 4 Department of Optometry, Mackay Junior College of Medicine, Nursing, and Management, New Taipei 11260, Taiwan; s458@eip.mkc.edu.tw; 5 School of Optometry, College of Medical Sciences and Technology, Chung Shan Medical University, Taichung 40201, Taiwan; syhuang0508@gmail.com (S.-Y.H.); vixa.zero@hotmail.com (J.-Z.C.); 6 Department of Medicine, Mackay Medical College, New Taipei 25245, Taiwan

**Keywords:** fucoxanthin, ultraviolet B, proinflammatory cytokine

## Abstract

Ultraviolet B (UVB) irradiation is the most common cause of radiation damage to the eyeball and is a risk factor for human corneal damage. We determined the protective effect of fucoxanthin, which is a carotenoid found in common edible seaweed, on ocular tissues against oxidative UVB-induced corneal injury. The experimental rats were intravenously injected with fucoxanthin at doses of 0.5, 5 mg/kg body weight/day or with a vehicle before UVB irradiation. Lissamine green for corneal surface staining showed that UVB irradiation caused serious damage on the corneal surface, including severe epithelial exfoliation and deteriorated epithelial smoothness. Histopathological lesion examination revealed that levels of proinflammatory cytokines, including tumor necrosis factor-α (TNF-α) and vascular endothelial growth factor (VEGF), significantly increased. However, pretreatment with fucoxanthin inhibited UVB radiation-induced corneal disorders including evident preservation of corneal surface smoothness, downregulation of proinflammatory cytokine expression, and decrease of infiltrated polymorphonuclear leukocytes from UVB-induced damage. Moreover, significant preservation of the epithelial integrity and inhibition of stromal swelling were also observed after UVB irradiation in fucoxanthin-treated groups. Pretreatment with fucoxanthin may protect against UVB radiation-induced corneal disorders by inhibiting expression of proinflammatory factors, TNF-α, and VEGF and by blocking polymorphonuclear leukocyte infiltration.

## 1. Introduction

Optical radiation includes ultraviolet (UV) radiation (200–400 nm), visible light (400–700 nm), and infrared radiation (700 nm–1 mm). Ambient UV radiation can be broadly divided into UVA (320–400 nm, approximately 90%) and UVB (290–320 nm, approximately 5%) wavebands. UVC (200–290 nm) is largely prevented from reaching the Earth’s surface by the ozone layer in the atmosphere [1]. UVB irradiation is mainly absorbed by the cornea and the anterior eye segment, through which the inner eye segments are protected from irradiation injuries. The cornea absorbs approximately 90% of UVB radiation and is highly sensitive to UVB damage [2]. Acute UVB exposure causes damage deeper than the corneal epithelium, thereby involving all corneal tissues, consequently inducing disorders in corneal cells [3]. The corneal damages caused by UV irradiation are collectively called photokeratitis or UV keratitis. The cellular and molecular mechanisms underlying photokeratitis have been extensively investigated [4,5,6]. Acute UVB irradiation induces various changes in DNA, proteins, and cells by activating proinflammatory mediators.

Inflammatory reaction to a multiplicity of insults includes the production of various cytokines and chemokines, such as tumor necrosis factor-α (TNF-α), which disrupts the barrier function and decreases the thickness of the corneal epithelium [7]. Ocular inflammation associated with ocular surface diseases affects corneal structure and function. The intensity of inflammation is associated with changes in the epithelium; for instance, inflammation is maximal when the corneal epithelium is disorganized [8]. Inflammation can be a potent trigger of corneal angiogenesis (or corneal neovascularization) [9]. Physiological balance of vascular biomarkers, such as vascular endothelial growth factor (VEGF), is crucial in the maintenance of normal vasculature [10]. An imbalance in angiogenic factors and related proteins may lead to proangiogenic processes. The early presence of infiltrated inflammatory polymorphonuclear leukocytes may act through cytokines to initiate the migration of keratocytes in the corneal stromal layer [11].

Fucoxanthin is an orange-colored pigment, along with chlorophylls a and c and β-carotene, present in brown seaweeds such as *Hijikia fusiformis*, *Laminaria japonica*, *Sargassum fulvellum*, and *Undaria pinnatifida* [12,13,14]. Brown seaweeds are the most common edible macroalgae in Southeast Asia and a few European countries. Brown seaweeds contain 4–10 g/kg fucoxanthin. The content of fucoxanthin is relatively high compared with the other carotenoids in natural products [15]. Fucoxanthin is metabolized to fucoxanthinol and amarouciaxanthin *in vivo* [16]. This pigment has noteworthy biological properties conferred by its inimitable molecular structure, which is analogous to neoxanthin, dinoxanthin, and peridinin, all of which differ from other carotenoids such as β-carotene. Fucoxanthin has therapeutic properties including antioxidant, anticancer, anti-inflammatory, anti-obesity, anti-diabetic, anti-angiogenic, and anti-malarial properties [17,18,19,20,21,22,23]. It also exerts protective effects on the liver, blood vessels of the brain, bones, and skin. However, the effects of fucoxanthin on light-induced damage in the corneal tissues of the eyeball have not been extensively examined.

To clarify the roles and underlying mechanisms of fucoxanthin at various stages of ocular lesion, we used a rat model to test the hypothesis that fucoxanthin ameliorates corneal damages caused by UVB irradiation. Male rats were treated with fucoxanthin daily, accompanied by UVB exposure for five days. Corneal surface damage was graded on the basis of corneal smoothness and extent of lissamine green staining. Corneal disorders were affiliated with thinning of the epithelial layer, stromal edema, and infiltration of polymorphonuclear leukocytes in the cornea. Furthermore, expressions of TNF-α and VEGF in the corneal tissues were observed to monitor corneal inflammation.

## 2. Results

### 2.1. Protective Effects of Fucoxanthin on UVB-Induced Corneal Disorders

**Figure 1:**
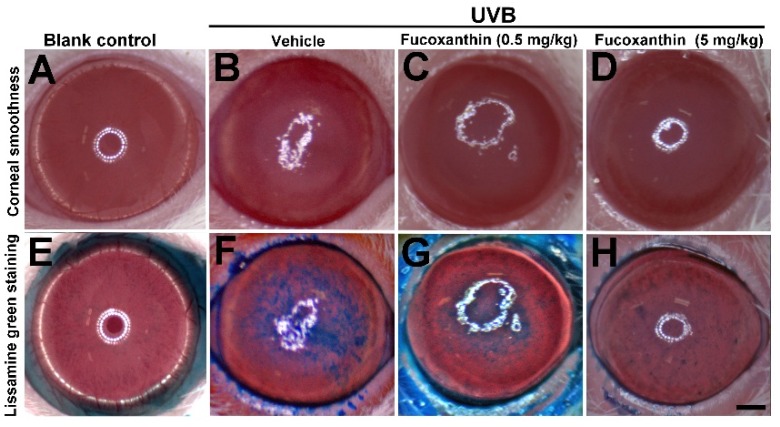
Effects of fucoxanthin on UVB-induced corneal damage. Corneal smoothness and lissamine green staining were compared with blank control (**A** and **E**), UVB/vehicle (**B** and **F**), UVB/fucoxanthin at 0.5 mg/kg (**C** and **G**), and UVB/fucoxanthin at 5 mg/kg (**D** and **H**) groups. UVB irradiation caused serious damage on the corneal surface (**B**), including severe epithelial exfoliation and deteriorated epithelial smoothness, as compared to blank controls (**A**). Analysis showed relative preservation of the intact corneal epithelium with fucoxanthin treatment at 0.5 and 5 mg/kg of body weight, respectively (**C** and **D**). With lissamine green staining, stained-epithelial areas on the ocular surface from UVB-induced damage was reduced in groups treated with 0.5 mg/kg (**G**) and 5 mg/kg fucoxanthin (**H**), when compared to the UVB/vehicle group; (**F**) Scale bar: 1 mm.

The corneal epithelium serves as a barrier that protects the eye from external agents, such as environmental hazards, and maintains corneal transparency. Superficial corneal analysis provided essential evidence of corneal disorders caused by UVB radiation. The corneas of the blank control rats were smooth, reflecting ocular surface integrity (Figure 1A). We observed irregular thickening and the distortion of irradiated corneas, mainly in the center, where the UVB radiation had been higher. UVB radiation caused severe injuries to the corneal epithelial surface (Figure 1B) including apparent corneal inflammation, severe epithelial exfoliation, and decomposed corneal smoothness. Although UVB radiation caused severe corneal epithelial injuries, UVB effects were inhibited by fucoxanthin. Significant improvement of the corneal surface was observed during examination of the groups treated with 0.5 and 5 mg/kg fucoxanthin (Figure 1C,D) compared with the corneal disorders detected in the UVB-treated experimental animals (Figure 1B).

Similar results were observed in the lissamine green-staining evaluation. Lissamine green staining largely remained unstained in the blank control group corneas (Figure 1E), whereas the dark-blue corneas compromised the integrity of the epithelial areas in the UVB-treated group (Figure 1F). Confluent to abundant punctate staining, large epithelial defects induced severe disorganization on the UVB-irradiated corneal surface. Compared with the injuries observed in the UVB-treated groups, smaller stained area (Figure 1G) and significantly fewer punctate-stained areas (Figure 1H) were observed on the ocular surface in fucoxanthin-treated groups at doses of 0.5 and 5 mg/kg, respectively.

**Figure 2:**
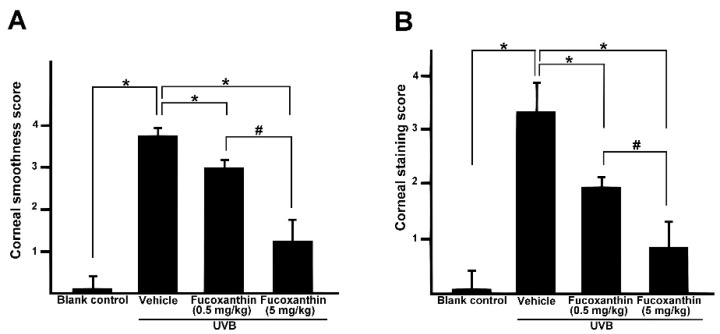
Effects of fucoxanthin on corneal surface irregularity index (**A**) and the lissamine green staining index (**B**) in UVB-induced corneal damage in rats. Data are shown as mean ± SD (*n* = 10 rats per group). * *p* < 0.05, compared to UVB/vehicle group, # *p* < 0.05, 0.5 mg/kg fucoxanthin group *versus* 5 mg/kg fucoxanthin group.

Semi-quantitative analyses of corneal smoothness and lissamine green staining were investigated and scored (Figure 2). All scores of cornea surface examination with statistically significant differences were observed between the UVB-treated group and those of the blank control group (*p* < 0.05), indicating that UVB irradiation triggered severe injury to the cornea. The corneas of the fucoxanthin-treated groups decreased the scores of corneal smoothness (Figure 2A) and lissamine green staining (Figure 2B) as compared to the UVB-treated group (*p* < 0.05), indicating that fucoxanthin treatment had reduced UVB-induced corneal disorders. Moreover, the analysis also revealed significantly decreased scores in the group treated with 5 mg/kg fucoxanthin as compared with the group treated with 0.5 mg/kg fucoxanthin (Figure 2A,B).

### 2.2. Inhibition of TNF-α and VEGF Activation Induced by UVB Irradiation with Fucoxanthin

**Figure 3:**
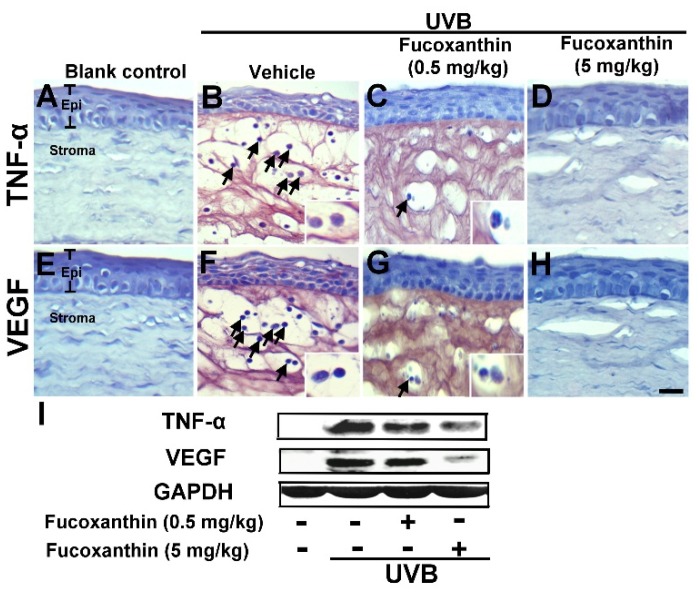
UVB-induced TNF-α (**A**–**D**) and VEGF (**E**–**H**) expression among blank control, UVB/vehicle, and UVB/fucoxanthin groups. Hematoxylin staining of corneas showed corneal epithelial exfoliation and decrease in thickness with UVB exposure (**B** and **F**) when compared to the blank control group (**A** and **E**) and the protective effect in UVB/fucoxanthin groups (**C**, **D**, **G** and **H**). Immunohistochemical staining showed evident induction of TNF-α and VEGF expression, and strong infiltration with polymorphonuclear leukocytes (arrows) in the UVB/vehicle group (**B** and **F**). In contrast, the experimental animals showed decreased expressions of TNF-α and VEGF in the epithelial layer (**C** and **G**) and in both epithelial and stromal layers (**D** and **H**) with fucoxanthin treatment at 0.5 and 5 mg/kg of body weight, respectively. Infiltrated leukocytes (arrow) are limited in corneas of UVB/ fucoxanthin groups (**C**, **D**, **G** and **H**). Moreover, Western blot analysis showed evident inhibition of TNF-α and VEGF expression in corneal tissues with 5 mg/kg fucoxanthin pretreatment groups (**I**). Epi, epithelium. Scale bar: 20 μm.

The irradiation of the anterior pole of the eye with UV caused significantly microscopic changes in all histological structures of the eye. To examine the effect of fucoxanthin on the UVB-induced disruption of corneal tissues, expressions of TNF-α (Figure 3A–D) and VEGF (Figure 3E–H) were measured with immunohistochemistry analysis. The analysis revealed a marked increase of TNF-α, which was induced by UVB exposure (Figure 3B), as compared to level of TNF-α in the blank control group animals (Figure 3A). The groups that received UVB irradiation showed significantly decreased corneal epithelial thickness when compared with the blank control group. Aside from the significant expression of TNF-α, surface desquamation, irregular surface, and loss of cell borders were observed in the UVB groups (Figure 3B). The corneal stroma was strongly infiltrated by polymorphonuclear leukocytes as a result of UVB irradiation. TNF-α expression and infiltrated leukocytes were intense at the center of the corneal stroma and correlated with the intensity of epithelial disruptions. The infiltration of polymorphonuclear leukocytes was highly significant when the epithelial lesions were at full intensity (Figure 3B). With UVB irradiation, the experimental animals 0.5 and 5 mg/kg fucoxanthin showed a decreased expression of TNF-α in the epithelium in the group treated with 0.5 mg/kg fucoxanthin (Figure 3C) and a significantly decreased expression of TNF-α in the epithelial and stromal layers in the group treated with 5 mg/kg fucoxanthin (Figure 3D). Moreover, the corneas of the fucoxanthin-treated groups showed a significantly lower number of infiltrated leukocytes (Figure 3C,D) than those of the UVB group (Figure 3B).

UVB irradiation enhanced intracorneal TNF-α expression was inhibited by fucoxanthin treatment. Therefore, we examined the effects of fucoxanthin on VEGF expression. VEGF, a proinflammatory cytokine, is upregulated in corneas with inflammation and is a significant angiogenic factor in corneal neovascularization in ocular inflammation. Histopathological evaluation revealed a significantly higher expression of VEGF in the UVB group (Figure 3F) than in the control group (Figure 3E). In contrast, both fucoxanthin treatment groups displayed less VEGF expression in the epithelial layer in the group treated with 0.5 mg/kg fucoxanthin (Figure 3G) or in epithelial and stromal layers in the group treated with 5 mg/kg fucoxanthin (Figure 3H) than the UVB group (Figure 3F). These findings suggest that the proinflammatory cytokines, including TNF-α and VEGF, being released in the cornea were effectively inhibited when treated with fucoxanthin. Moreover, Western blot analysis indicated evident inhibition of TNF-α and VEGF expression with fucoxanthin pretreatment, respectively (Figure 3I).

### 2.3. Effect of Fucoxanthin on the Thickness of Corneal Epithelial and Stroma Layers

**Figure 4:**
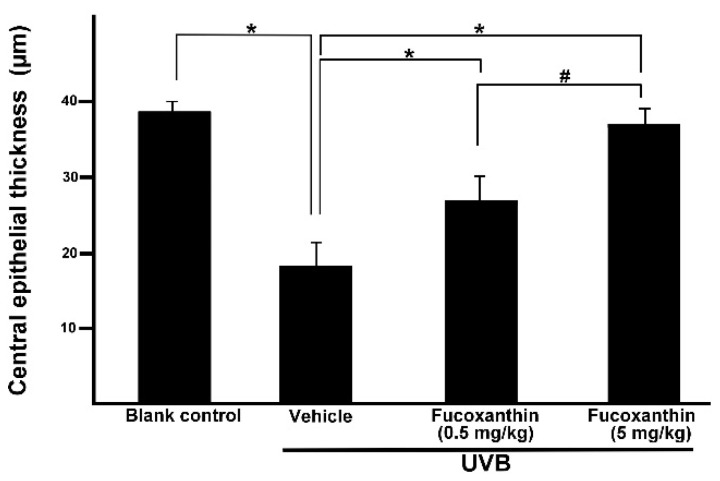
Effects of fucoxanthin on central epithelial thickness in UVB-induced corneal injury in rats. Data are shown as mean ± SD (*n* = 10 rats per group). * *p* < 0.05, compared to the UVB/vehicle group, # *p* < 0.05, 0.5 mg/kg fucoxanthin group *versus* 5 mg/kg fucoxanthin group.

To confirm whether fucoxanthin administration affected corneal morphological changes, we measured the thickness of the cornea. After UVB irradiation, thinning of the corneal epithelial layer was observed in the eyes of the rats. The present study revealed that fucoxanthin induced significant preservation of the corneal epithelial thickness in both fucoxanthin-treatment groups (26.5 ± 3.4 μm for 0.5 mg/kg group; 36.5 ± 1.8 μm for 5 mg/kg group) compared with the vehicle-given group (17.9 ± 3.1 μm) after UVB irradiation. Significant improvement of central epithelial thickness was detected during examination in the group treated with 5 mg/kg fucoxanthin as compared with corneal thickness detected in the group treated with 0.5 mg/kg. In addition, corneal thickness remained similar to that of the naive controls (38.4 ± 1.2 μm) in the rats treated with 5 mg/kg fucoxanthin (Figure 4).

**Figure 5:**
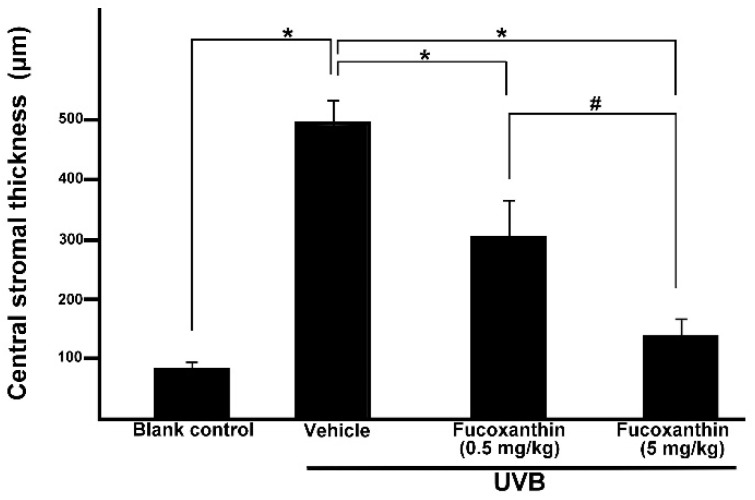
Effects of fucoxanthin on central stromal thickness in UVB-induced corneal injury in rats. Data are shown as mean ± SD (*n* = 10 rats per group). * *p* < 0.05, compared to the UVB/vehicle group, # *p* < 0.05, 0.5 mg/kg fucoxanthin group *versus* 5 mg/kg fucoxanthin group.

Considering the corneal epithelial deficiency in the fucoxanthin-treatment groups after UVB irradiation, we conducted further pathologic examinations to analyze the thickness of stromal tissues. Accumulation of inflammatory cells and angiogenesis blood vessels contribute to the thickness of the corneal stroma [8]. In the present study, significantly thicker corneal stroma tissues were observed in the UVB group (495.2 ± 37.3 μm) than in the blank control group (83.3 ± 9.6 μm). Statistical analysis showed significantly decreased thickness of stromal edema in both fucoxanthin-treated groups (306.7 ± 58.1 μm for 0.5 mg/kg group; 136.2 ± 26.5 μm for 5 mg/kg group) when compared with that in the vehicle-treated group after UVB exposure (495.2 ± 37.3 μm, Figure 5). Rats treated with 5 mg/kg fucoxanthin revealed a significant reduction in the stromal edema as compared with the group treated with 0.5 mg/kg fucoxanthin. On the basis of these observations, we conclude that treatment with fucoxanthin served as possible protection from UVB radiation-induced inflammatory disorders in experimental rats by inhibiting the expression of inflammatory factors such as TNF-α and VEGF.

## 3. Discussions

The corneal epithelium is the first protector of optic axis changes with irregular thickening and distortion of UVB radiation exposure, mainly at the center, which is where the UVB radiation was high in the present study. UVB irradiation initiates molecular responses in the cornea primarily by triggering excessive generation of reactive oxygen species (ROS), which contribute to inflammatory cytokines [24]. In clinical diagnosis and investigational analysis, lissamine green staining is commonly used to evaluate ocular surface disorders because of its high sensitivity and specificity.

Analysis of the superficial cornea (the corneal surface) provided essential evidence on corneal disorders caused by UVB radiation. We demonstrated that fucoxanthin inhibited UVB-induced disorders in the corneal tissues. Fucoxanthin exerts protective effects on UVB-induced expression of inflammatory cytokines and infiltrated polymorphonuclear leukocytes in the cornea. The expression levels of TNF-α and VEGF, which contributed to ocular inflammation, were attenuated by fucoxanthin in the corneal epithelium. These results suggest that fucoxanthin inhibited UVB-induced disruption of barrier function in corneal epithelial cells. Furthermore, fucoxanthin ameliorated corneal epithelial damage and preserved corneal epithelial thickness. Fucoxanthin also appears to protect the corneal epithelium from disruption of barrier function *in vivo*.

Previous studies have indicated that fucoxanthin treatment prevents epidermal cells from UVB and blue light irradiation, possibly through antioxidant and anti-inflammatory effects in the skin *in vivo* and *in vitro* [25,26]. The cornea develops from both surface ectoderm and mesoderm. Hence, the cornea is associated with many skin diseases as well as systemic, metabolic, and immune diseases. Our results showed that fucoxanthin treatment suppresses UVB-induced TNF-α and VEGF expression in corneal tissues. The suppressive effects of fucoxanthin on TNF-α and VEGF may be important in avoiding corneal injuries including photokeratitis.

Corneal epithelial abrasion can result in stromal edema *in vivo* [27]. Previous studies also indicated that VEGF is upregulated in corneal stroma (infiltrated leukocytes and resident corneal cells) during inflammatory response and is involved in injury-induced neovascularization [28,29,30,31]. In the present study, aside from anterior epithelial lesions, intense inflammatory reaction was also observed in the stromal layer, with the expression of the inflammatory cytokines TNF-α and VEGF, numerous of infiltrated polymorphonuclear leukocytes, and stromal edema. The corneal thickness may be changed after fixation and embedding, but the significant difference of relative thickness could be still observed. The present study indicated that the intensity of the inflammatory response in the stroma correlated with the changes in the epithelium, where the anterior epithelium was swelling and damaged, and inflammation was maximal.

A previous study demonstrated that preventing potential angiogenesis by treatment with fucoxanthin can be useful in preventing angiogenesis-related diseases such as cancer, diabetic retinopathy, atherosclerosis, and psoriasis [17]. Other studies showed that fucoxanthin significantly decreases the expression of VEGF in sarcoma 180 of xenografts-bearing mice in a dose-dependent manner *in vivo* [32]. The corneal surface examination in the present study is the first to demonstrate that fucoxanthin treatment ameliorated UVB radiation-induced corneal damage, while also down-regulating VEGF expression. The results of these studies agree in that fucoxanthin exhibits potent anti-angiogenic activity.

Inflammatory reaction, a self-defensive response against numerous pathogenic stimuli, is categorized by attracting large aggregates of leukocytes and may become a harmful self-damaging process. Fucoxanthin had been previously determined to reduce the levels of proinflammatory mediators, including NO, PGE_2_, IL-1β, TNF-α, and IL-6, by inhibiting NF-κB activation and suppressing mitogen-activated, protein kinases phosphorylation in lipopolysaccharide-induced macrophage RAW264.7 cells [33]. In the present study, the corneas of the fucoxanthin-treated groups showed significantly inhibited expression and decreased amount of infiltrated leukocytes compared with those of the UVB group. The swelling of inflammatory response in the stroma correlated with the expression and decreased numbers of infiltrated leukocytes in the stroma. In contrast, we demonstrated that fucoxanthin treatment reduced the inflammatory infiltrate and ameliorated stromal edema in UVB-induced corneal disorders.

Experimental studies have shown a number of polymorphonuclear leukocytes in the aqueous humor through the cornea irradiated with UVB and footpad injected with lipopolysaccharide [34,35]. In the present study, experimental animals were treated with 5 mg/kg fucoxanthin, which significantly reduced cellular infiltration in the stroma of cornea. We suggest another possibility for the protective mechanism of fucoxanthin: inhibition of the breakdown of the blood-aqueous-barrier and the depression of infiltration of the iris-ciliary leukocytes in the aqueous humor by UVB-induced disorders to the ocular anterior segments.

Carotenoids play an important role in protecting cells and organisms against the harmful effects of light, air, chemicals, and oxidative stress. Previous studies suggested that topical administration of astaxanthin suppressed phototoxicity in the corneal epithelium [36]. Other study has demonstrated that *D. salina*, which contains abundant carotenoids, increases antioxidant enzyme activity and prevents corneal oxidative stress at doses of 123 and 615 mg/kg [37]. In the present study, we demonstrated that treatment with 5 mg/kg fucoxanthin inhibited the expression of proinflammatory factors, reduced the inflammatory infiltrate, and ameliorated stromal edema in UVB-induced corneal disorders. Treatment with fucoxanthin may, therefore, be a candidate for treatment that limits damage due to UV irradiation.

## 4. Experimental Section

### 4.1. Experimental Animals

Male Sprague–Dawley rats (4–5 weeks old, 200–300 g) were obtained from the Animal Department of BioLASCO Taiwan (Taipei City, Taiwan). The experimental animals were quarantined and allowed to acclimate for a week before the experiment. The animal room temperature was maintained at 25 ± 2 °C and the relative humidity at 55% ± 5%. Air handling units in the animal room were set to provide approximately 12 fresh air changes per hour. Three to four animals were housed per cage under standard laboratory conditions, with a 12-h light/dark cycle. Care and treatment of the animals were in accordance with the standard laboratory animal protocols approved by the Animal Care Committee (Mackay Medical College, New Taipei, Taiwan). Food and water were available *ad libitum*. The experimental protocols for this study were approved by the Institutional Animal Care and Use Committee and the animals were handled in accordance with institutional ethical guidelines.

### 4.2. UVB-Induced Corneal Disorders and Fucoxanthin Treatment

**Figure 6:**
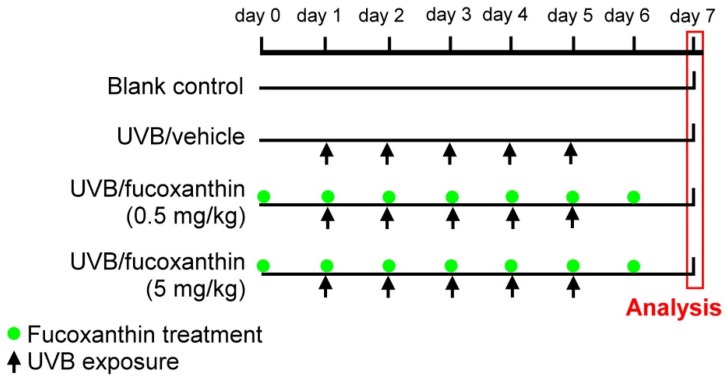
Experimental protocol for UVB irradiation and fucoxanthin treatment. Daily UVB irradiation (indicated by arrows) was performed from day 1 to day 5. The UVB/vehicle group served as UVB controls exposed to UVB light and pretreated with an intravenous injection of 0.1% dimethyl sulfoxide solution mixed with 0.1 mL PBS daily for seven days (day 0 to day 6). UVB/fucoxanthin groups were exposed to UVB irradiation and pretreated intravenously with 0.5 or 5 mg/kg fucoxanthin daily (indicated by green point) for seven days (day 0 to day 6).

A total of 40 rats were randomly divided into four groups: Group I: blank control, Group II: UVB/vehicle (exposure to UVB irradiation and pretreatment with intravenous injection of 0.1% dimethyl sulfoxide solution (Sigma-Aldrich, St. Louis, MO, USA) mixed with 0.1 mL phosphate-buffered saline (PBS), Group III: UVB/fucoxanthin (exposure to UVB irradiation and pretreatment with intravenous injection of 0.5 mg/kg fucoxanthin (Sigma-Aldrich) in 0.1% dimethyl sulfoxide solution mixed with 0.1 mL PBS), and Group IV: UVB/fucoxanthin (exposure to UVB irradiation and pretreatment with intravenous injection of 5 mg/kg fucoxanthin in 0.1% dimethyl sulfoxide solution mixed with 0.1 mL PBS). To induce disorders in the corneal tissues, the eyes of the animals in groups II, III, and IV were exposed to UVB irradiation following a previously described protocol [38] with slight modifications. The energy output of UVB was measured with an UV detector (USB2000+UV-VIS, Ocean Optics, Dunedin, FL, USA) from 200–850 nm. The wavelength of the light source peaked at 310 nm (range, 280–315 nm). After anesthesia with intraperitoneal injection of sodium pentobarbital (60 mg/kg body weight), the eyes were exposed to 550 μW/cm^2^ of UVB light (UVM-28; UVP Inc., San Gabriel, CA, USA) for 3 min in a darkroom. The entire UVB irradiation course was completed within five consecutive days (day 1 to day 5, Figure 6). Group II (UVB/vehicle) animals, which served as UVB controls, were exposed to UVB irradiation with intravenous injection of 0.1% dimethyl sulfoxide solution mixed with 0.1 mL PBS. The animals in groups III and IV (UVB/fucoxanthin) were exposed to UVB irradiation and intravenously pretreated with 0.5 and 5 mg/kg fucoxanthin (1 day before UVB exposure) daily for seven days (day 0 to day 6, Figure 6), respectively.

### 4.3. Determination of Corneal Disorders

The corneas were observed under a stereoscopic microscope (Stemi DV4 Stereo Microscope, Carl Zeiss, Oberkochen, Germany). To acquire the image of the cornea, a circular illuminator source was attached to the stereoscopic microscope. Swamp irregularity, which reflects corneal surface integrity, was quantified following a previous protocol [38] with slight modifications to evaluate corneal surface irregularities caused by UVB-induced damage. Corneal surface irregularity was graded on a scale of 0–4 as follows: grade 0, absent; grade 1, mild; grade 2, moderate; grade 3, severe with twist circle shape; and grade 4, severe with undistinguished shape.

After scoring corneal smoothness, the eyes were stained with 1% lissamine green (Sigma-Aldrich, St. Louis, MO, USA) and then washed thrice with 0.9% saline. Previous carcinogenicity and toxicity studies were unremarkable and demonstrated an excellent safety profile [39]. Ocular surface examination with an instillation of 1% lissamine green had good inter-observer reliability and was well-tolerated. Images of lissamine green staining of the corneal surface were obtained and scored in accordance with the following grading system based on stained proportion in the damaged cornea [40]: grade 0, the total area without dot staining; grade 1, less than 25% of the cornea stained with dotted punctuate staining; grade 2, 25%–50% of the cornea stained with diffuse punctuate staining; grade 3, 50%–75% of the cornea stained with punctate staining and apparent epithelial defects; grade 4, more than 75% of the cornea stained with profuse punctate staining and large epithelial defects. The final numerical score was calculated by dividing the sum of the number per grade of the affected rats by the total number of examined animals.

### 4.4. Histopathological Analysis and Immunohistochemistry of Inflammatory Reaction in the Cornea

Experimental rats were anesthetized with choral hydrate (400 mg/kg of body weight, intraperitoneally) and perfused with 4% paraformaldehyde. Sagittal paraffin-embedded sections of 8 μm were cut with a rotary microtomes, placed on glass slides, and allowed to dry at room temperature for 15 min. After being deparaffinized and rehydrated, the sections were transferred into PBS solution. For immunohistochemistry, the tissue sections were boiled in citrate buffer (pH 6.0) for 20 min for antigen retrieval, incubated in 2% hydrogen peroxide to eliminate endogenous peroxidase activity, and blocked using 5% normal goat serum and 0.5% Triton X-100 in PBS. The sections were incubated overnight at 4 °C with goat anti-TNF-α (1:200, sc-1350; Santa Cruz, Dallas, CA, USA) and rabbit anti-VEGF (1:100, A-20; Santa Cruz, Dallas, CA, USA) polyclonal antibodies to detect the inflammatory reaction of TNF-α and VEGF expression, respectively. The slides were incubated with secondary biotin-conjugated anti-goat or anti-rabbit immunoglobulin antibodies (1:200; Sigma-Aldrich, St. Louis, MO, USA). The immune complexes were detected using an ABC kit and a 3,3-diaminobenzadine Substrate Kit (Vector Labs, Burlingame, CA, USA) in accordance with the manufacturer’s instructions. The slides were then counterstained with hematoxylin and analyzed under a Zeiss Axiophot microscope (Carl Zeiss, Oberkochen, Germany).

### 4.5. Western Blot Analysis

Corneal tissues were rinsed once with ice-cold PBS and then lysed with PBS containing 1% Triton X-100, 0.1% SDS, 0.5% sodium deoxycholate, 1 μg/mL leupeptin, 10 μg/mL aprotinin and 1 mM phenylmethylsulfonyl fluoride on ice for 20 min. After using an electric homogenizer, crude extracts were subjected to centrifugation at 4 °C. The supernatants were collected as tissue lysates. All protein concentrations were determined by a protein assay (Bio-Rad laboratories, Richmond, CA, USA). Aliquots (50 μg) of tissue lysates were separated electrophoresed on 8 SDS-polyacrylamide gel and then transblotted onto the ImmobilonTM-P membrane. After being blocked with 10% skim milk in Tween-20/PBS, blots were incubated with TNF-α, VEGF, and GAPDH (sc-32233; Santa Cruz, Dallas, CA, USA) antibodies and then incubated with HRP-conjugated secondary antibodies. The protein bands in the blots were detected using enhanced chemiluminescence kit (ECL; Bio-Rad laboratories). The visualization of GAPDH was used to ensure equal sample loading in each lane. All experiments were repeated at least three times.

### 4.6. Corneal Thickness Measurements

After being deparaffinized and rehydrated, the corneal sections were stained with hematoxylin. The sections were considered as the central cornea by the presence of the pupil and the optic nerve. For each cornea, separate perpendicular measurements, laterally separated by 50 μm, were made from the basement membrane of the epithelium to the top of Decemet’s membrane and from the basement membrane of the epithelium to the top of the epithelium at the central region of the corneal cross section. The mean measurement was reported as the central stromal and epithelial thickness of the cornea [41]. The thickness of the cornea was determined using image analysis software (Image-Pro Plus v. 4.5, Media Cybernetics, Silver Spring, MD, USA).

### 4.7. Statistical Analysis

Means and standard deviations were calculated for all parameters determined in this study. Comparison analyses between any two groups were performed using Student’s *t*-test. Statistically significant differences between groups were defined as *p* < 0.05.

## 5. Conclusions

Treatment with fucoxanthin protected from UVB radiation-induced corneal disorders, including corneal surface irregulation, thinning of the epithelial layer, stromal edema, and infiltration of polymorphonuclear leukocytes, in animal experiments. Our observations demonstrated that the protective capacities of fucoxanthin may be attributed to the decrease in the expression of inflammatory cytokines TNF-α and VEGF. Fucoxanthin may be useful as a protective agent against UVB radiation-induced corneal damage *in vivo*.
